# Hemotympanum as a Complication of a Valsalva Maneuver during Childbirth

**DOI:** 10.1155/2023/3328895

**Published:** 2023-08-09

**Authors:** Tali Teitelbaum, Isaac Shochat, Golda Grinblat, Mohamad Taha, Itzhak Braverman

**Affiliations:** Hillel Yaffe Medical Centre, Technion Faculty of Medicine, Hadera, Israel

## Abstract

**Background:**

Hemotympanum may occur due to otic barotrauma secondary to Valsalva maneuver during the second phase of labor. A pressure differential across the tympanic membrane (TM) of about five psi can cause rupture. The increased intrathoracic and intraabdominal pressure spikes repeatedly manifested by “pushing” during second-stage labor easily approach (and may exceed) this level. *Clinical Presentation*. This case report describes a healthy thirty-seven-year-old multipara patient admitted for the 40-weeks' gestational age routine follow-up that proceeded to active labor followed by an aural fullness and bloody otorrhea. Otoscopic examination with a light microscope confirmed the hemotympanum of the right tympanic membrane.

**Conclusion:**

Forceful Valsalva can cause hemotympanum. Investigating the benefits and disadvantages of the pushing methods could help reduce such complications in the future. A prompt evaluation of an otolaryngologist should be requested in the event of a new postpartum hearing disturbance or bloody otorrhea.

## 1. Background

Hemotympanum is defined by the presence of blood in the middle ear cavity that corresponds to purple discoloration or ecchymosis of the TM.

A common cause of hemotympanum is barotrauma [1]. During active vaginal delivery, the woman is “pushing the baby out,” performing repeated Valsalva maneuvers [2] (further referred to as Valsalva), which can eventually increase middle ear pressure and cause barotrauma. Perforation of the TM due to Valsalva has been reported in association with delivery [3]. Described here is a case of labor-induced hemotympanum, which has not been reported in the English literature to the best of our knowledge.

Hemotympanum can occur for various reasons such as temporal bone fracture due to blunt trauma, fracture of the external auditory canal, ossicular trauma, middle ear barotrauma [4], therapeutic nasal packing, epistaxis [5, 6], blood disorders, anticoagulation therapy, and otitis media.

Hemotympanum presents as the presence of blood in the middle ear cavity or ecchymosis of the TM, which leads to conductive hearing loss, thus the need to perform an audiogram. Patients with hemotympanum commonly complain of otalgia, tinnitus, and aural fullness [1].

The management of hemotympanum is conservative, but antibiotics may be prescribed for infection prophylaxis.

### 1.1. Clinical Presentation

An otherwise healthy, thirty-seven-year-old multipara, G3P2, presented to the emergency ward at 40 + 0 gestational weeks in active delivery. Her pregnancy was remarkable for an increased alpha-fetoprotein and an abnormal glucose challenge test; however, amniocentesis, a brain-directed fetal ultrasound (US), and a glucose tolerance test were found normal. She had no recorded history of otolaryngologic procedures or complaints and no recent barometric pressure changes.

During the obstetric examination, her vital signs, aside from elevated blood pressure, were unremarkable, with a low Bishop score (a prelabor scoring system assisting in predicting whether labor induction would be required). The fetal parameters, including weight and movements, confirmed by the fetal US, were within normal limits. Due to gestational age, multiparity, and elevated blood pressure with patient consent, membrane-stripping was performed to induce labor.

The patient preferred avoiding labor augmentation and was transferred to the delivery room to support natural childbirth. The delivery proceeded uneventfully, without anesthesia or analgesia, until reaching a normal third stage. Without a need for an episiotomy, a healthy boy has been delivered, weighing 4234 grams with an APGAR score of 9/10. The placenta was delivered spontaneously and was intact. The patient has received routine postpartum uterine stimulants.

After childbirth, the patient noticed aural fullness and hearing loss in her right ear. Shortly after noticing these symptoms, she was referred to the otolaryngology resident for prompt examination. During anamnesis, she denied earache, tinnitus, or dizziness. During the examination, spontaneous right-ear firm otorrhea was seen, like a blood clot blocking the medial portion of the external canal. No previous head trauma or complaints of concurrent earache, facial nerve palsy, tinnitus, or dizziness were registered. Since the resident assumed that the otorrhea was a blood clot, a hydrogen peroxide solution toilette was initiated and suctioned to clear the external ear canal. After several attempts to clean the external ear canal, the microscope otoscopy exam still revealed that the medial part of the external ear canal was obstructed. For this reason, she was advised to repeat the exam the following day if her symptoms did not improve.

**Figure 1:**
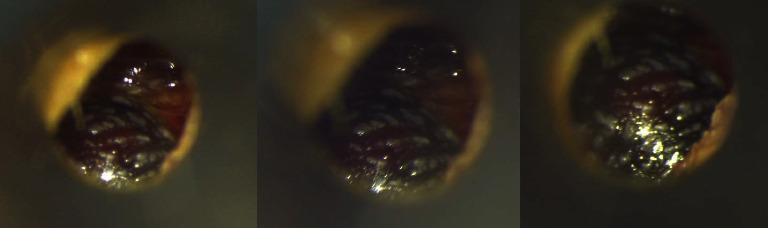
Micro-otoscopy showing postpartum hemotympanum.

On the follow-up exam, the patient was examined by a different ENT resident. The patient complained only of aural fullness. Microscope otoscopy displayed a bulgy purple mass covering the whole diameter of the TM without noticing perforation or fresh bleeding (Figure 1). After a thorough examination and consultation, complete with an anterior rhinoscopy and right-leaning Weber examination, she was diagnosed with hemotympanum secondary to childbirth. The patient was instructed to perform a complete audiogram and reevaluation the next day; however, she did not return. Further documentation from a community follow-up showed no additional reports of a hemotympanum.

## 2. Discussion

The TM is a 1 cm diameter half-transparent, oval-shaped membrane which separates the external ear from the middle ear. It consists of three layers: the outer layer stratified squamous keratinized epithelium; the middle layer lamina propria; and the inner layer simple cuboidal epithelium. The lamina propria contains the blood vessels of the tympanic membrane [7].

In 1963, Thornhill described the hemotympanum as a “blue eardrum” since it was caused by the presence of blood in the middle ear [8]. The definition of hemotympanum is the presence of blood in the middle ear cavity that corresponds to purple discoloration or ecchymosis of the TM. Common causes of hemotympanum are temporal bone fractures, therapeutic nasal packing, epistaxis, blood disorders, anticoagulant therapy, otitis media, and barotrauma.

While no similar cases of labor-induced hemotympanum were found in the English written literature, Baum et al. reported a TM perforation following the intrapartum Valsalva maneuver [3].

A possible causing mechanism could be ear barotrauma, created by unequalized pressure differentials between the middle and external ears [9].

Ear barotrauma can be caused during flying, diving, and aggressive Valsalva [6, 9], a forceful attempt of acceleration against a closed airway, performed to equalize pressure across the TM and open the Eustachian tubes during changes in the ambient pressure.

When barotrauma occurs, as in aggressive Valsalva, the created vacuum leads to an increase in the blood flow of the subcutaneous vessels of the TM and blood engorgement, which can cause serous effusion. Should the pressure continue to rise without equalization of the middle ear, it will result in traumatic injury to the TM and middle ear and consequent rupture of the TM blood vessels into or behind the TM, also known as hemotympanum.

Attention is needed following hemotympanum since the accumulation of blood in the middle ear may lead to conductive hearing loss (CHL). Hemotympanum is managed conservatively [10], but antibiotics may be prescribed for infection prophylaxis. However, Becker and Parell note that prophylactic antibiotics and decongestants have not been proven helpful in the case of barotrauma [11].

While mostly harmless, hemotympanum might lead to ossicular chain disruption or fixation, mainly of the IS joint, due to residue formation, hyalinization, as well as ischemic necrosis of the long process of the incus, as it is a watershed area. While no reported study has been performed on these theorized connections, it might be prudent to follow-up with audiological and otological examinations.

The forceful Valsalva maneuver also affects the inner ear, which may lead to persistent hearing loss due to inner ear hemorrhage, tinnitus due to an intracochlear membrane tear, and dizziness due to a perilymph fistula [12].

The Eustachian tube is an essential component of ambient pressure equalization. Its dysfunction is common during pregnancy due to the increased mucosal edema, causing obstruction and otitis media with effusion [13, 14]. Thus, pregnancy may lead to ear pressure function abnormalities and predisposition to barotrauma.

The second stage of labor, in which the cervix is fully dilated, is considered the pushing stage, which is critical for the baby's head to navigate through the maternal pelvis. In this stage, the woman is guided to perform the Valsalva pushing maneuver by taking a deep breath at the beginning of the contraction, holding her breath (closed glottis), and pushing as long and as hard as she can, along with uterine contractions [15]. This contrasts with the “physiological pushing” when the woman directly pushes in response to an involuntary urge with an open glottis without taking a deep breath first [2].

Caldeyro-Barcia postulated [16] that closed glottic pushing influences maternal hemodynamics and increases intrathoracic pressure. However, during exhalation with an open glottis, air escapes, and the thoracic pressure is not maintained. The valveless venous system of the head and neck allows for direct transmission of intrathoracic or intraabdominal pressure into the head and neck. As described by Gibran et al.[17], the sudden elevation of venous pressure may cause instant rupture of superficial retinal capillaries and decompensation in the retinal capillary bed, with subinternal limiting membrane hemorrhages.

Similarly, in the current case report, the Valsalva pushing maneuver during the second stage of delivery led to an increased intrathoracic or intraabdominal pressure with a consequent elevation of the venous pressure in the head and neck areas, presumably causing the rupture of the tympanic membrane capillaries and the ecchymoses. The baby's size could also play a role in such an event, as in the present case, the newborn was 4234 g which is above the 95th percentile weight for male babies [18] and might have led to a greater effort while pushing.

Other possible explanations include masked coagulation disorder, a vascular cause such as an arterial aneurysm, as described by Horowitz et al. [19], or tympanomastoid or jugular paragangliomas [20], that both can lead to a hemorrhage and bloody otorrhea. In addition, while not probable, it could be that the hemotympanum was an unrelated occurrence.

## 3. Conclusions

Pushing during labor can affect remote and small areas of our body, such as the retina and the tympanic membrane. It is not clear if our patient used the Valsalva pushing or the physiological pushing, but either way, it was forceful enough to cause hemotympanum, as described here. A prompt evaluation of an otolaryngologist should be requested in the event of a new postpartum hearing disturbance or bloody otorrhea.

## Data Availability

Any additional information can be obtained by contacting the main author.
